# Feasibility of trauma-focused cognitive behavioral therapy for traumatized children in Japan: a Pilot Study

**DOI:** 10.1186/s13033-015-0021-y

**Published:** 2015-07-03

**Authors:** Satomi Kameoka, Junko Yagi, Yoko Arai, Sachiko Nosaka, Azusa Saito, Wakako Miyake, Saeko Takada, Sayaka Yamamoto, Yasuko Asano, Eizaburo Tanaka, Nozomu Asukai

**Affiliations:** Hyogo Institute for Traumatic Stress, 1-3-2 Wakinohama-kaiganndori, Chuo-ku, Kobe, Hyogo 651-0073 Japan; Department of Neuropsychiatry, Iwate Medical University, 19-1 Uchimaru, Morioka-shi, Iwate 020-8505 Japan; Victim Support Center of Tokyo, 3-18-1, Toyama, Shinjuku-ku, Tokyo, 169-0052 Japan; Graduate School of Human Sciences, Osaka University, 1-2 Yamadaoka, Suita, Osaka 565-0871 Japan; Faculty of Human Sciences, Mejiro University, 4-31-1, Naka-Ochiai, Shinjuku-ku, Tokyo, 161-8539 Japan; Osaka Medical Center and Research Institute for Maternal and Child Health, 840 Murodo-cho, Izumi, Osaka 594-1101 Japan; Osaka Prefectural Child Guidance Center, 28-5 Yasaka-tyou, Neyagawa, Osaka 572-0838 Japan; Tokyo Metropolitan Institute of Medical Science, 2-1-6 Kamikitazawa, Setagaya-ku, Tokyo, 156-8506 Japan

**Keywords:** Children, Post-traumatic stress disorder, Trauma, Trauma-focused cognitive behavioral therapy, Treatment

## Abstract

**Background:**

Trauma-focused cognitive behavioral therapy is used to treat children who have experienced traumatic events and suffer from trauma-related disorders. Its effectiveness has been demonstrated in several randomized controlled studies. However, most of these studies have been performed in the United States, with few studies conducted in Asian countries. Therefore, we aimed to evaluate the feasibility of trauma-focused cognitive behavioral therapy in children who have experienced traumatic events and who suffer from trauma-related disorders in Japan.

**Findings:**

Thirty-five traumatized children (mean age = 10.9 years; range = 3–17 years; 74.3% girls) who received trauma-focused cognitive behavioral therapy were included. The effectiveness of the program was evaluated in each case using the University of California at Los Angeles Post-Traumatic Stress Disorder Reaction Index for DSM-IV for trauma-related symptoms and the Children’s Global Assessment Scale for social functioning. Pre- and post-treatment outcome measures were analyzed using two-tailed paired *t* tests. The results for 35 participants indicate that post-traumatic stress symptoms were significantly improved following therapy [t(35) = 8.27; p < 0.01], whereas the assessment of social functioning supported the effectiveness of the program [t(35) = −14.68; p < 0.01]. The pre- to post-treatment effect sizes (Glass’s delta) were 1.24 for the University of California at Los Angeles Post-Traumatic Stress Disorder Reaction Index and 1.96 for the Children’s Global Assessment Scale.

**Conclusions:**

Our findings indicate that trauma-focused cognitive behavioral therapy is feasible for treating traumatized children of an Asian population. We discuss the implications of this result for clinical practice and future research.

## Background

The number of children in Japan that suffer trauma is increasing. For example, the annual number of consultations at child guidance centers for child abuse and neglect increased approximately 60-fold over the past 20 years in Japan. Furthermore, the Great East Japan Earthquake and Tsunami of March 11, 2011 resulted in 1,698 children experiencing parental death, of which 241 were orphaned. These traumatized children require emotional and practical support, and sometimes professional treatment, to prevent the future adverse mental health or behavioral problems.

Trauma-focused cognitive behavioral therapy (TF-CBT) is a treatment that includes both the parent and child [1], with a strong evidence base in several randomized controlled trials (RCTs) in the US [2–7]. These studies have demonstrated the efficacy of TF-CBT in reducing post-traumatic stress (PTS) symptoms and other emotional problems in children. Additional RCTs of TF-CBT have been reported outside the US in Australia (sexually abused children [8]), the Democratic Republic of Congo (former child soldiers [9] and sex-trafficked girls [10]), and Norway (children with mixed trauma [11]). Although dissemination is ongoing in several other countries, we are not aware of any research on TF-CBT in Asian countries that has provided sufficient guidance for evidence-based practice.

In 2011, we began implementing TF-CBT for traumatized children and their caregivers in Japan, using a version identical to the original US version without any culture-related modification. In this uncontrolled pilot study, we show the feasibility of TF-CBT in Japan.

## Method

### Participants and procedures

Between June 2012 and February 2014, participants were recruited from four sites in Japan: a child-trauma center in Morioka, a trauma care center in Kobe, a victim support center in Tokyo, and a child guidance center in Osaka. We recruited children with mixed trauma between the ages of 3 and 17 years and their caregivers. For inclusion in the study, children were required to meet at least five criteria for post-traumatic stress disorder (PTSD) as defined in the Diagnostic and Statistical Manual of Mental Disorders, Fourth Edition (DSM-IV), including at least one symptom in each of the three PTSD clusters (re-experiencing, avoidance, and hyperarousal). In addition, children were required to have a caregiver who was willing and able to participate every week in the parental treatment included in TF-CBT. Informed child assent and parental consent were required for admission to the study. We excluded patients with psychosis, history of suicide attempt, or any significant behavioral problems, including those with aggressive and/or antisocial behaviors that cause significant impairment in social, academic, or other important areas of functioning. Child psychiatrists or clinical psychologists screened 187 children and identified 35 children and their caregivers to be eligible for treatment in this study. Among these participants, 3 dropped out during the treatment; 32 children completed all TF-CBT components. Of these, 74.3% (n = 26) of the children were girls and their mean age was 10.9 years [standard deviation (SD), 3.6]. Most cases of trauma were because of sexual abuse inside or outside the family (42.9%, n = 15). Table 1 summarizes the demographic data, type of traumatic experience, and relationship between child and caregiver for the participants.

**Table 1:** Description of sample

Demographics of child sample (*N* = 35) and participating caregivers (*N* = 35)	*n* (%)
Gender (*N* = 35)	
Girls	26 (74.3)
Boys	9 (25.7)
Age (*N* = 35)	
Range	3–17
Mean	10.9
SD	3.6
Most traumatic experience—total (*N* = 35)	
Natural disaster (earthquake and/or tsunami)	9 (25.7)
Sexual abuse outside the family	10 (28.6)
Sexual abuse inside the family	5 (14.3)
Physical abuse inside the family	5 (14.3)
Bullying	3 (8.6)
Kidnap	2 (5.7)
Traffic accident	1 (2.9)
Total number of trauma experiences (*N* = 35)	
Range	1–4
Mean	1.8
SD	0.8
Identity of participating caregiver (*N* = 35)	
Biological mother	16 (45.7)
Biological father	2 (5.7)
Both biological mother and father	8 (22.9)
Grandmother	2 (5.7)
Aunt	2 (5.7)
Nursery home careworker	5 (14.3)

### Therapists

Therapists delivered the treatment at each site. They comprised three child psychiatrists and four clinical psychologists. All therapists were women and underwent a 2-day introductory training session by the program developers and/or were certified TF-CBT trainers.

### TF-CBT

The TF-CBT program uses a component-based cognitive behavioral approach over 12–25 sessions. Each component, as shown in Table 2, is offered to the child and caregiver in both parallel and joint sessions [1]. In this study, all sessions were discussed with peer consultation and supervised by the first author, and 30% of the sessions were audio recorded. In 33% of the sessions, ongoing consultation was provided by the program developers or a certified trainer. All sessions were coded using the Brief Practice Checklist (BPC) and were confirmed to meet fidelity standards by a certified TF-CBT trainer. The BPC, created by the program developers, tracked the timing and implementation of specific TF-CBT components to help therapists and supervisors determine whether fidelity was adequately maintained.

**Table 2:** PRACTICE components

Psychoeducation about child trauma and trauma reminders
Parenting component including parenting skills
Relaxation skills individualized to youth and parent
Affective modulation skills tailored to youth, family and culture
Cognitive coping: connecting thoughts, feelings and behaviors
Trauma narrative and processing
In vivo mastery of trauma reminders
Conjoint youth-parent sessions
Enhancing safety and future developmental trajectory

### Measures

The traumatic events and PTS symptoms of children were evaluated using the University of California at Los Angeles PTSD Reaction Index for DSM-IV (i.e., the RI) [12], which is a widely used, well-validated, and reliable measure. The RI is a child- and parent-reported questionnaire that evaluates the exposure to traumatic events and all DSM-IV PTS symptoms that occurred between ages 7 and 18 years. This test has been translated by Akashi et al. and examined for reliability by Fujimori et al. [13] in Japan. In this study, the child scores were adopted and analyzed without the data for a 3-year-old girl; the score for her was provided by her mother only.

The Children’s Global Assessment Scale (C-GAS) was used to assess the social functioning of the children [14]. The C-GAS is a well-validated and reliable measure that is equivalent to the adult Global Assessment of Functioning score; we used its Japanese version in this study [15]. TF-CBT therapists conferred with an independent evaluator to determine each child’s C-GAS score. This independent evaluator decided the final C-GAS score by reviewing each child’s social functioning, but was not involved in the treatment.

### Ethical considerations

This study was approved by the institutional review board of each institute or facility. Furthermore, after the study procedure had been completely explained to them, written assent and consent to participate were obtained from children and their caregivers, respectively.

### Statistical analysis

Paired *t* tests were performed to determine the pre- and post-treatment effect. Two-tailed *p* values <0.05 were considered statistically significant. Intention-to-treat (end-point) analyses were performed in which pretest scores were used to replace missing post-test scores. Effect sizes for the two primary outcomes were calculated using Glass’s delta.

## Result

The number of TF-CBT sessions for those who completed the program ranged from 9 to 26 [mean (*M*) = 14.31; *SD* = 3.60]. The length of the sessions ranged from approximately 60 to 90 min in this study.

The pre- and post-treatment means and standard deviations are presented in Table 3. Pre- and post-treatment outcome measures were analyzed using two-tailed paired *t* tests. The post-treatment RI scores (*M* = 12.69; *SD* = 11.46; *range* = 0–26) were significantly lower than the pretreatment RI scores (*M* = 29.06; *SD* = 13.21; *range* = 6–56; *t* = 8.27; *p* < 0.01) for the 35 children that were assessed. Furthermore, post-treatment C-GAS scores (*M* = 73.74, *SD* = 11.48, *range* = 61–91) were significantly higher than pretreatment C-GAS scores (*M* = 53.31, *SD* = 10.43, *range* = 38–70; *t* = −14.68; *p* < 0.01) for the 35 children that were assessed. The RI and the C-GAS scores were well correlated pretreatment (r = −0.66; *p* < 0.01) and post-treatment (r = −0.74; *p* < 0.01). The Cronbach’s alpha for the RI was 0.85 (n = 35) pretreatment, and 0.80 (n = 31) post-treatment.

**Table 3:** Descriptions of outcome variables: means and standard deviations by treatment time

Measure	Pre-treatment		Post-treatment		p	Effect size
	M	SD	M	SD		Pre–post
RI (*N* = 35)	29.06	13.21	12.69	11.46	<0.01	1.24
CGAS (*N* = 35)	53.31	10.43	73.74	11.48	<0.01	1.96

*RI* UCLA PTSD Index for DSM-IV, *CGAS* Children’s Global Assessment Scale, *M* mean, *SD* standard deviation, *N* number of cases, *Effect size* glass’s delta.

A reliable cut-off value for a single incident traumatic event in the English version of the RI is considered to be 38 [12]. In this sample, 25.7% of the participants were above this level and 74.3% were below this level on their pretreatment RI scores. Of those who completed all components of the program, 71.9% (n = 23) exhibited at least a 50% reduction in their RI score. Though only one child experienced an increase in their post-treatment RI score (pre–post; 12–17), her post-treatment C-GAS score was higher than her pretreatment score (pre–post; 55–65). The pre- to post-treatment effect sizes (Glass’s delta) were 1.24 for RI and 1.96 for the C-GAS.

The characteristics of the three children who dropped out were as follows: a 10-year-old girl who experienced sexual abuse by a stranger, a 13-year-old boy who experienced an earthquake and sexual abuse, and a 17-year-old girl who experienced physical abuse. Their pretreatment RI scores were 19, 40, and 56, and their pretreatment C-GAS scores were 70, 45, and 38, respectively. They dropped out at a session of 20, 8, and 7, respectively.

## Discussion

In this pilot study, we found that TF-CBT was feasible for children suffering from PTS symptoms because of mixed trauma in Japan. PTS symptoms and general social functioning significantly improved in participants by the end of treatment, with considerable effect sizes: 1.24 and 1.96 for RI and C-GAS, respectively.

The pre–post effect sizes of our study were smaller than those of previous TF-CBT studies in the West, which are as follows: they range from 2.13 to 2.80 using the Kiddie Schedule for Affective Disorders and Schizophrenia in the US studies for sexually abused children [3, 5, 16]; 1.92 using the Child PTSD Symptom Scale in a Norwegian study of children subjected to mixed traumatic events [11]; and 1.86 using the Anxiety Disorder Interview Schedule, Child Version, in an Australian study for sexually abused children [8]. Although these differences may be caused by the different assessment scales used, it was noteworthy that most therapists in our study were being trained and that their treatment skills were consequently immature. Furthermore, 60% of participants in our study were victims of single trauma, such as crime or the Great East Japan Earthquake and Tsunami. Therefore, we assume that the total number of trauma experiences in our study (*M* = 1.8) was markedly lower than that of the studies in the US, Norway, or the Democratic Republic of Congo (*M* = 3.56–11.79) [7, 10, 11].

Three children failed to complete the TF-CBT program because they showed strong resistance to creating or processing trauma narratives. Moreover, one of those patients had the highest RI score in our study. We believe that the further experience and skill acquisition by our therapists will help overcome this issue in the future.

In the original version of TF-CBT, therapists are encouraged to modify their interventions specific to each child. However, the requirement for culture-specific modifications to the treatment program has been discussed when seeking to disseminate TF-CBT to non-English speaking countries. Although culture-specific modification was not required for the implementation of TF-CBT in Norway [11], dissemination in low-resource African countries required modifications because of cultural differences and the limited availability of trained therapists [9, 10, 17, 18]. We found that no culture-related modifications were required in Japan, suggesting that TF-CBT may be successfully applied according to the original US version in Asian settings.

There are several limitations to this study. First, this was an uncontrolled pilot study in Japan. Therefore, the effectiveness of TF-CBT has not yet been established. Second, we measured treatment outcomes with a self-rating questionnaire of PTS symptoms and the therapist’s evaluation of social functioning. Although this could cause a bias in the results, the self-rating and therapist-rating scores were well correlated before and after treatment. Third, the therapists received ongoing consultation in one-third of cases by program developers or a certified trainer in the US because it was a part of their TF-CBT training. Therefore, ongoing consultation probably contributed to the success observed.

Despite the limitations of the study, we believe that our preliminary findings demonstrate the feasibility of TF-CBT in Japan. Moreover, they suggest that TF-CBT in Japan is a promising treatment for traumatized children and their caregivers. Following this study, a randomized-controlled trial with independent assessments is required to evaluate the effectiveness of TF-CBT for traumatized children in Japan.
